# The combined effect of physical activity and fruit and vegetable intake on decreasing cognitive decline in older Taiwanese adults

**DOI:** 10.1038/s41598-022-14219-5

**Published:** 2022-06-14

**Authors:** Richard Szewei Wang, Bing-Long Wang, Yu-Ni Huang, Thomas T. H. Wan

**Affiliations:** 1 Affiliation Program of Data Analytics and Business Computing, Stern School of Business, New York University, New York, 10012 USA; 2 School of Health Policy and Management, Chinese Academy of Medical Sciences and Peking Union Medical College, Beijing, 100730 China; 3 College of Public Health, National Taiwan University, 100 Taipei, Taiwan; 4 School of Global Health Management and Informatics, University of Central Florida, Orlando, FL 32816 USA

**Keywords:** Health care, Health policy, Health services, Public health

## Abstract

The factors associated with cognitive decline among older adults include physical activity and fruit and vegetable intake. However, the long-term effects of concomitant physical activity and fruit and vegetable intake are unknown. This 16-year longitudinal study explored the joint effect of mitigating cognitive decline in a cohort of older Taiwanese individuals. Five population-based surveys (Taiwan Longitudinal Survey on Aging [1999–2015]) involving 4440 respondents over 53 years old in 1999 were conducted. Cognitive function was assessed using the Short Portable Mental Status Questionnaire (SPMSQ). The demographic, socioeconomic, health-related, behavioral, and disease status covariates were adjusted in the regression analysis. Trends in cognitive decline were observed over 16 years. The risk of cognitive decline decreased by 63% when high physical activity and high fruit and vegetable intake were combined (odds ratio 0.37; 95% confidence interval 0.23–0.59), indicating a potential combined effect of physical activity and fruit and vegetable intake on mitigating cognitive decline. These personal actions are safe, effective, and economical approaches to health promotion and disease prevention.

## Introduction

The global population is aging rapidly. The population over the age of 65 years is estimated to increase by 12% (approximately 1 billion) by 2030 and 16.7% (approximately 1.6 billion) by 2050^1^. As older individuals live longer, the most common cause of cognitive impairment is dementia and has become a major public health problem^2^. In 2018, approximately 31 million adults aged ≥ 50 years were inactive (i.e., they had no physical activity [PA] beyond daily living)^3^. Low levels of PA can contribute to heart disease, type 2 diabetes, cancer, obesity, and cognitive impairment^4^. In addition to PA, diet plays an important role in the health of elderly individuals. Nutritional problems are common among individuals over 60 years of age. The prevalence of nutritional deficiency in older adults living in communities ranges from 1.3 to 47.8%^5^. The relationship between nutritional deficiencies, as risk factors, and cognitive decline has been documented by Scholey and Gonzalez, et al.^6,7^. Cognitive decline can cause loss of independence and decreased quality of life in older adults^8^. According to the Alzheimer’s Association in the US, the medical expenditure owing to cognitive decline for the population over 65 years of age in 2020 was US $30.5 billion and is expected to exceed US $1.1 trillion by 2050^2^.

Cognitive decline in older adults causes severe social and economic problems. Previous studies have identified PA as an effective mechanism to delay cognitive decline^9^. For example, PA effectively delays cognitive decline, and high PA has a better protective effect on cognitive decline than low PA^10,11^. For older adults, PA has apparent health benefits, including improvement of cognitive function and reduction of the risk of Alzheimer’s disease and neuropsychiatric symptoms^12–15^. Lack of PA is one of the many risk factors for cognitive decline among elderly individuals^16^. Simple PA in daily life, such as walking, also benefits their health and cognitive function^17,18^.

Intake of polyphenol-rich foods, such as fruits and vegetables, can be an alternative of vitamins or supplements^6,19^. Fruits and vegetables may be the leading food groups for improving cognitive function^20^, since dietary factors play a crucial role in preventing cognitive decline, as documented in National Institutes of Health studies^21^. Many studies have confirmed the association between diet and cognitive function^22–24^. Elderly individuals who consume a large amount of fruits and vegetables have a significantly reduced risk of experiencing mild cognitive decline^25^ Previous studies have also shown an association between Mediterranean-style diet patterns and specific nutrients against cognitive decline^26–30^.The factors associated with cognitive decline among older adults include PA^17,18^ as well as fruit and vegetable (F&V) intake separately^21,22^. However, the long-term effects of concomitant PA and F&V intake are unknown. Herein, we hypothesized that joint high PA and high F&V intake are associated with the decrease in cognitive decline in adults-in-midlife and older Taiwanese. Our study suggests that the provision of specific and easy-to-implement diet and exercise recommendations is essential to the promotion of well-being in their daily lives.

## Methods

### Data and sample

Data were obtained from the Taiwan Longitudinal Survey on Aging (TLSA). This project was jointly implemented by the Taiwan Health Promotion Administration and the Population Studies Center of the University of Michigan and funded by the US National Institute on Aging and the government of Taiwan^31^. The TLSA survey aimed to determine the impact of demographic, socioeconomic, environmental, and lifestyle changes on older Taiwanese health, healthcare use, and cognitive status. A three-stage proportional probability sampling technique was adopted, and the sampling frame was based on the household registration data. Trained interviewers conducted face-to-face interviews. The TLSA uses measurement scales in population-based research and is known for its high completion rate and acceptable data quality^32^. Detailed information on the study design and sampling of the TLSA has also been previously described by Tsai et al. and Zimmer et al.^33,34^.

**Figure 1:**
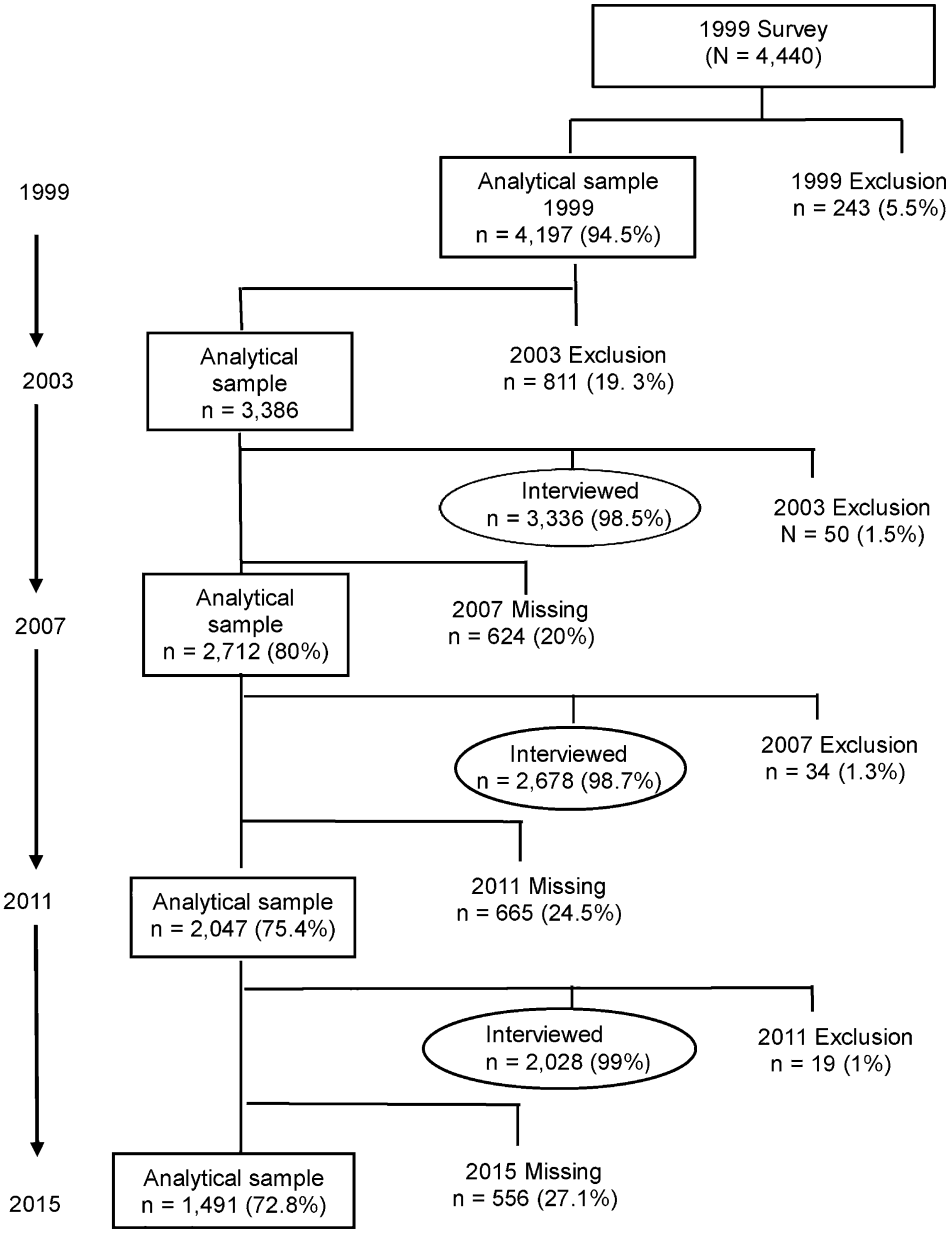
Participants in the Taiwan Longitudinal Survey on Aging from 1999 to 2015. Missing cases were treated as incomplete data for major constructs.

This study explored longitudinal trends in cognitive status using five-wave population-based surveys conducted over a 16-year period (1999–2015). The 1999 survey contained data on physical activity and fruit and vegetable measures, and was therefore selected as the baseline for our study. Data from the 1999 (baseline), 2003, 2007, 2011, and 2015 surveys were used, and a categorical variable was created to distinguish the data. During the follow-up period, the numbers of missing and dead individuals increased. The number of respondents in each year was as follows: 4197 (in 1999), 3386 (in 2003), 2712 (in 2007), 2047 (in 2011), and 1491 (in 2015) respondents who completed five-wave interviews and self-reported data were included in the analysis (Fig. 1). The TLSA protocol was reviewed and approved by government-appointed representatives, and the study was conducted in accordance with the ethical standards outlined in the Helsinki Declaration. All participants in the panel provided informed consent.

This study analyzed a panel of middle-aged and older adults from 1999 to 2015. In conducting a panel/longitudinal study, a starting age group of 50 years was selected in 1996. Therefore, this cohort population began at 53 years of age in 1999^33,34^. Of the 4400 eligible older adults, we excluded individuals with missing data for either the Chinese version of the Short Portable Mental Status Questionnaire (SPMSQ) or sensory decline items. Specifically, we excluded 243 individuals with cognitive decline defined as SPMSQ scores of ≤ 6 during their initial assessments in 1999. Such exclusion of older adults with cognitive decline constituted the panel of elders for a prospective study and ensured the accuracy and validity of the responses in the initial assessments. Ultimately, 1491 older adults without cognitive decline in the baseline (Time 1) were included in the analysis.

### Outcome measure

The SPMSQ, developed by E. Pfeiffer, has 10 items^35^. Each item is given a score of 0 for no error or 1 for an error. Assessment of orientation to time, person, and place generates the total number of errors. Higher error scores indicate poorer cognitive function. Specifically, 0–2 error scores indicate normal functioning; 3–4 error scores, mild cognitive impairment; 5–7 error scores, moderate cognitive impairment; and ≥ 8 error scores, severe cognitive impairment. The total adjusted scores range from 0 to 10, with higher scores indicating poorer cognitive function. One more error was allowed in the scoring when the participants had had a grade school education or less. An additional minor error was allowed when the participants had an education beyond the high school level^36^. The participants with two or more errors from each former wave survey score were described as having cognitive decline^37^.

### Independent variables

PA frequency, duration and intensity, which were adopted from the World Health Organization, Haase et al., and Pitsavos et al., were measured^38–40^. The following three questions were included: (i) “How often do you engage in routine PA?” The choices provided were (a) none (invalid), (b) less than twice a week, (c) three to five times a week, and (d) more than or equal to six times a week. (ii) “How many minutes do you spend each time?” The choices provided were (a) < 15 min/time, (b) 15–30 min/time, and (c) ≥ 30 min/time; (iii) “After you exercise, do you sweat or gasp?” The choices provided were (a) no sweating or gasping and (b) some or much sweating or gasping. The scores for the three questions were multiplied to obtain the PA score for each participant. The total score ranged from 0 to 18. PA was divided into three levels: low (total score = 0), moderate (total score = 1–7), and high (total score = 8–18). Herein, PA did not include work-related activities or activities of daily living; its reliability is generally acceptable^40,41^.

F&V intake was evaluated using a validated semiquantitative questionnaire that assessed the frequency of intake of food categories. The frequency of F&V intake was calculated as follows: every day or almost every day as six times, three to five times a week as four, once to twice a week as 1.5, less than once a week as 0.5, and zero times a week as 0; the total number was divided into three groups: low (< 7 times a week), moderate (7–9 times a week), and high (≥ 10 times a week).

PA and F&V intake were combined and divided into five groups: low (no PA and weekly F&V intake for < 7 times), both high (high PA and weekly F&V intake for ≥ 10 times), only high PA (high PA and weekly F&V intake for < 7 or 7–9 times), only high F&V intake (F&V intake for ≥ 10 times per week and no or moderate PA), and others (low PA and/or F&V intake for 7–9 times per week).

### Covariates

Time-varying covariates of several indicators of health behaviors and concurrent health indicator were included in adjusted logistic regression analysis to determine the net influence of PA and F&V intake on cognitive decline. The education adjustment score was applied to three groups: ≤ 6, 7–12, and ≥ 13 years. The participants were divided into two groups according to alcohol consumption: never drank and drank once or more a week (yes vs. no); smoking: non-smoker or current and past smokers (yes vs. no); and tea consumption: three or more times and less than three times a week. Chronic diseases, such as hypertension, diabetes, heart disease, stroke and cancer, were determined according to whether a physician had told the respondents that they had the disease. Depressive symptoms were measured using the Depression Short Form (CES-D-10)^31,42^, a 10-item Likert-scale questionnaire assessing depressive symptoms based on self-reports during the past week. The total summative scores of the items range from 0 to 30. A score of ≥ 10 was considered indicative of depressive symptoms. The life satisfaction index (LSI)^43^ (total score range: 0–10) was used to assess life satisfaction; an LSI of ≥ 6 was considered indicative of life satisfaction^41,44^.

### Data analysis

All statistical analyses were performed using SPSS version 22.0. Generalized estimating equations (GEE) with robust standard error estimates were used to consider within-subject correlations (i.e., autocorrelations) during the 16-year follow-up period^45,46^; data from the 1999 (baseline), 2003, 2007, 2011, and 2015 five-wave follow-up interviews were assessed simultaneously in all analyses. Baseline measures of cognitive status were included to reduce unobserved heterogeneity. The longitudinal models included measures of cognitive status from previous waves to examine the associations among PA, F&V intake, and subsequent cognitive status. Given the considerable changes in socio-economic status, family structure, and physical health at the end of the life course, we assessed the robustness of their associations over time. Multiple time-varying attributes of the study participants were included in the multivariate analysis to adjust for variability. Thus, the net influences of PA and F&V intake on cognitive decline were determined.

## Results

Table 1 presents the essential characteristics of the dataset. The baseline number of participants in 1999 was 4,197. More men (51.0%) than women (49.0%) were included. The majority of the participants were 53–64 years old (61.2%). Approximately 75.2% had formal education for ≤ 6 years, while 7.0% had education for > 13 years. The men had less cognitive decline than the women (11.4% vs. 22.8%). Most of the participants reported being married (79.2%), not smoking (78.6%), and not drinking (72.0%). More than a quarter (27.8%) of the participants were diagnosed with hypertension. Other chronic diseases reported included diabetes (8.0%), heart disease (14.6%), stroke (1.7%), cancer (2.1%), and depressive symptoms (12.6%). The majority of the participants felt satisfied with life (64.0%). Over half of the men (53.1%) were classified into the high PA group. The proportion of women was higher than that of men in the low PA group. The ≥ 75-year-old group with high PA and the 53–64-year-old group with high F&V intake had the highest proportions (65.3% and 80.9%). When we combined the PA and F&V intake groups, the 65–74-year-old group with both high parameters showed the highest proportion (49.0%). Among the participants in the ≥ 13 educated-years group, 15.2% reported low PA; this proportion was lower than that in the other two education groups; furthermore, the highest proportion was observed in the high PA (64.8%), high F&V intake (94.3%), and combined PA and F&V intake groups (61.9%). Regardless of whether the participants were married, the proportion of low PA was more than one-third (39.4% and 39.0%). The high PA group had the most married participants (49.8% and 47.1%); the married participants had a higher proportion in the three following groups: high F&V intake, both high, and only F&V high. The proportion of smokers was higher in the low PA group (45.5%) than in the moderate and high PA groups. More than half of the participants in the high PA (50.9%), high F&V intake (83.6%), and both high groups (41.9%) were non-smokers. Approximately 51.9% of the participants in the drinking group had a high PA compared with 48.2% of those in the non-drinking group. Moreover, the proportion of participants in the drinking group with a high F&V intake was lower. The participants in the high PA, high F&V intake, and both high groups (55.2%, 85.7%, and 48.3%, respectively) felt satisfied with their life. Those who reported being dissatisfied with life had a higher proportion of cognitive decline at baseline (1999) than their counterpart. Chronic diseases were not significantly associated with cognitive decline among the older Taiwanese individuals.

**Table 1:** Participant characteristics at baseline of the Taiwan Longitudinal Survey on Aging (1999).

		Physical activity (%)			F&V intake (%)			Combined physical activity and F&V intake (%)					Cognitive decline (%)
		Low	Moderate	High	Low	Moderate	High	Both low	Both high	Only high PA	Only high FV	Other	Yes
**Sex (%)**													
Men	51.0	35.7	11.2	53.1	10.9	11.4	77.7	5.3	42.7	8.5	34.7	8.8	11.4
Women	49.0	42.8	11.7	45.5	7.9	9.8	82.3	3.6	37.6	4.9	44.3	9.6	22.8
**Age (%)**													
53–64	61.2	46.3	12.6	41.1	8.5	10.7	80.9	4.5	34.6	5.2	45.9	9.8	13.6
65–74	32.1	29.1	9.6	61.3	10.7	8.8	80.4	4.2	49.0	7.9	31.0	7.9	22.8
≧ 75	6.8	24.8	9.9	65.3	10.9	17.8	71.3	5.0	47.5	13.9	23.8	9.9	22.8
**Education (%)**													
≦ 6	75.2	45.3	10.2	44.5	10.9	12.4	76.7	5.2	34.2	7.3	42.2	11.2	19.4
7–12	17.8	23.4	13.6	63.0	5.7	5.7	88.6	2.3	56.6	5.7	31.7	3.8	7.5
≧ 13	7.0	15.2	20.0	64.8	1.9	3.8	94.3	1.9	61.9	1.9	32.4	1.9	17.1
**Marriage (%)**													
Yes	79.2	39.4	10.8	49.8	8.5	9.9	81.6	3.9	40.8	6.5	40.5	8.3	15.6
No	20.8	39.0	13.9	47.1	12.7	13.0	74.4	6.5	37.4	7.1	36.5	12.6	23.2
**Smoking (%)**													
Yes	21.4	45.5	11.6	42.9	17.0	15.8	67.2	10.3	33.5	10.0	33.2	12.9	10.7
No	78.6	37.6	11.4	50.9	7.3	9.2	83.6	2.8	41.9	5.7	41.4	8.2	18.9
**Drinking (%)**													
Yes	28.0	35.4	12.7	51.9	13.3	9.9	76.9	6.2	41.6	10.0	34.7	7.4	10.8
No	72.0	40.8	11.0	48.2	7.9	10.8	81.3	3.7	39.5	5.3	41.6	9.9	19.7
**Hypertension (%)**													
Yes	27.8	29.7	13.0	57.2	8.0	9.4	82.6	2.4	47.1	6.5	35.3	8.7	17.9
No	72.2	43.0	10.9	46.1	9.9	11.0	79.1	5.2	37.4	6.7	41.3	9.4	16.9
**Diabetes (%)**													
Yes	8.0	31.7	16.7	51.7	7.5	8.3	84.2	3.3	42.5	1.7	41.7	10.8	23.3
No	92.0	40.0	11.0	49.0	9.5	10.8	79.7	4.5	39.9	7.1	39.5	9.0	16.6
**Heart disease (%)**													
Yes	14.6	33.6	16.6	49.8	9.8	12.1	78.1	6.0	41.5	6.0	35.9	10.6	19.8
No	85.4	40.3	10.6	49.1	9.3	10.3	80.4	4.2	39.9	6.8	40.3	8.9	16.7
**Stroke (%)**													
Yes	1.7	44.0	8.0	48.0	12.5	4.2	83.3	8.0	36.0	8.0	44.0	4.0	12.0
No	98.3	39.2	11.5	49.2	9.3	10.7	80.0	4.4	40.2	6.6	39.6	9.3	17.3
**Cancer (%)**													
Yes	2.1	25.0	18.8	56.3	12.5	3.1	84.4	3.1	50.0	3.1	34.4	9.4	15.6
No	97.9	39.6	11.3	49.1	9.3	10.7	80.0	4.5	39.9	6.7	39.8	9.2	17.2
**Tea consumption (%)**													
< 3	64.1	40.7	11.8	47.5	10.3	10.7	79.1	4.3	37.5	6.7	41.3	10.1	19.5
≧ 3	35.9	36.8	10.7	52.4	7.7	10.5	81.8	4.3	44.7	6.6	37.0	7.3	13.2
**Depressive symptoms (%)**													
No	87.4	37.3	10.0	52.7	7.9	10.2	81.8	3.5	43.5	7.0	38.1	8.0	15.7
Yes	12.6	48.3	17.8	33.9	21.4	10.4	68.2	10.3	23.0	6.9	44.8	14.9	25.3
**Life satisfaction (%)**													
Un-satisfied	36.0	45.7	11.9	42.3	14.3	14.1	71.6	7.7	30.6	8.7	40.5	12.6	21.7
Satisfied	64.0	34.4	10.3	55.2	6.8	7.5	85.7	2.3	48.3	5.5	37.3	6.7	14.9

**Figure 2:**
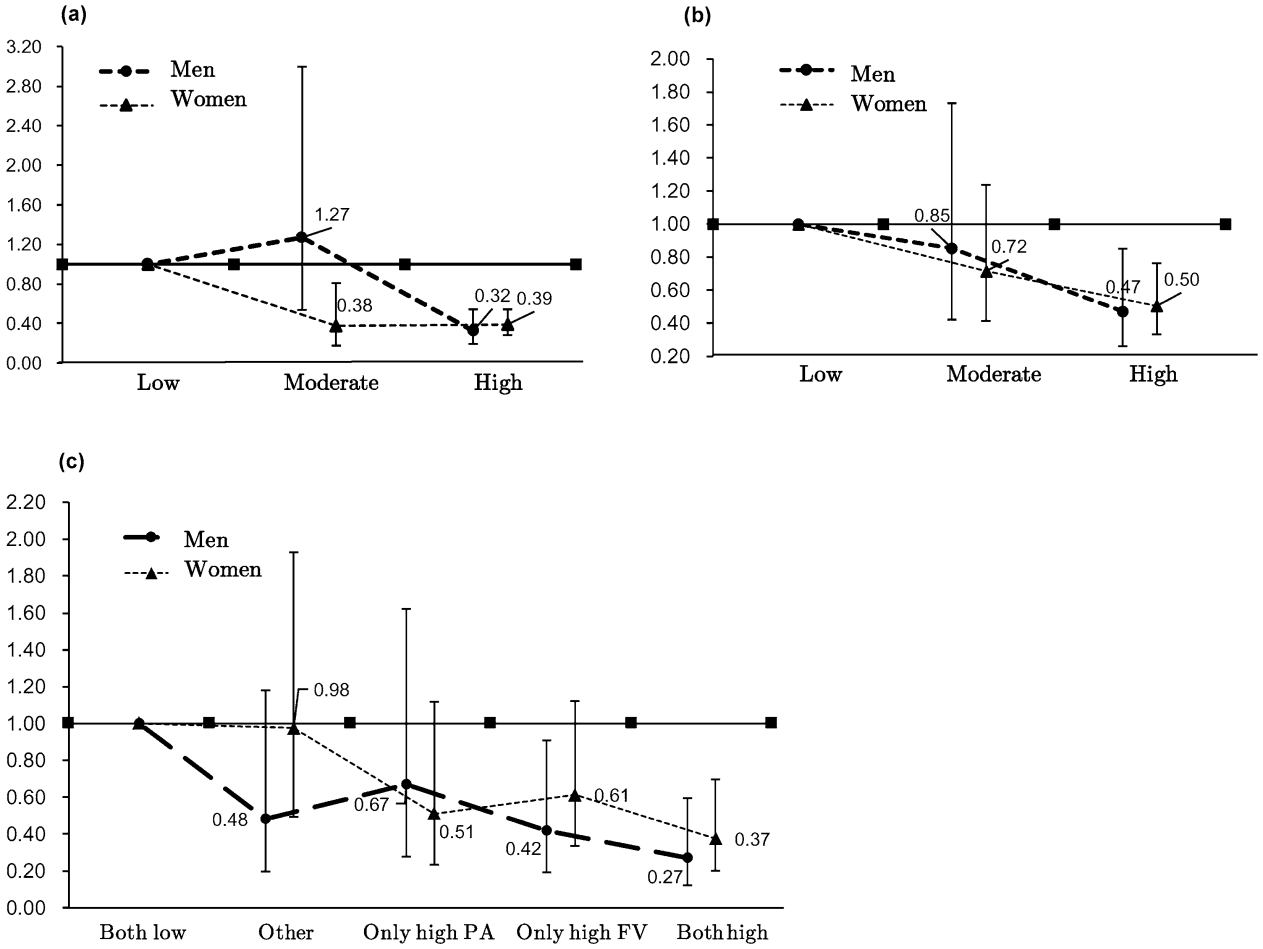
Differences in (**a**) physical activity, (**b**) fruit and vegetable intake, and (**c**) combined physical activity and fruit and vegetable intake according to sex. Bars show the 95% confidence intervals.

Figure 2a shows a significant difference in the multiple odds ratio (OR) (p < 0.01). The OR among the women was 0.39 (p < 0.01), and the multiple OR among the men was 0.32 (p < 0.01), indicating greater effects of decreasing cognitive decline in the men than in the women. The high and low F&V intake groups showed significant differences in cognitive decline. In these groups, the OR among the women was 0.50, and the OR among the men was 0.47 (p < 0.01), as shown in Fig. 2b. Figure 2c shows that the combination of the high PA and high F&V intake groups and combination of the low PA and low F&V intake groups showed multiple significant differences in cognitive decline (p < 0.01). The combined OR among the women was 0.37–0.98. The effects were better among the men than among the women (OR: 0.27–0.48).

Table 2 presents the impact of PA and F&V intake at the reference point on the cognitive decline of older Taiwanese after 16 years, and GEE regression was adopted. Sex, age, education, marriage, smoking, drinking, hypertension, heart disease, diabetes, cancer, stroke, CES-D score, and LSI were statistically controlled. In Model 1, significant results were obtained (p < 0.05), with ORs of 0.40 and 0.58, indicating that moderate and high PA can decrease cognitive decline by 60% and 42%, respectively. Moreover, a high F&V intake was associated with a decrease in cognitive decline by 40%. A moderate F&V intake was associated with a decrease in cognitive decline by 0.86 folds. Although the difference was not statistically significant (p = 0.202), the trend was consistent. Model 2 presents the effects of combining PA and F&V intake on cognitive decline during the 16 years of follow-up. Compared with that in the both low group, the prevalence of cognitive decline in the both high group decreased by 63% (p < 0.00); only high F&V group, 45% (p < 0.00); only high PA group, 40%; and others group, 23%; these results showed no statistical significance (p > 0.05). The results suggest that both groups (intake of F&V more than 10 times per week combined with PA for less than twice a week, 15–30 min/time) and the only high F&V intake group achieved a decrease in cognitive decline.

**Table 2:** Adjusted longitudinal associations of physical activity and F&V intake with cognitive decline in the Taiwan Longitudinal Survey on Aging from 1999 to 2015.

	Model 1		Model 2	
	OR	95% CI	OR	95% CI
**Physical activity**				
High	0.40	0.30–0.52		
Moderate	0.58	0.32–1.02		
Inactive	1			
**Fruit-vegetable intake**				
High	0.60	0.41–0.84		
Moderate	0.86	0.53–1.37		
Low	1			
**Combine physical activity (PA) and fruit-vegetable (F & V) intake**				
Both high			0.37	0.23–0.59
Only high PA			0.60	0.34–1.05
Only high FV			0.55	0.35–0.87
Others			0.77	0.44–1.33
Both low			1	
**Sex**				
Men	1		1	
Women	0.55	0.40–0.74	0.55	0.41–0.75
**Age**				
53–64	1		1	
65–74	11.75	5.38–25.63	11.71	5.41–25.32
≧ 75	33.91	15.63–73.57	36.94	17.20–79.29
**Education (years)**				
≦ 6	1		1	
7–12	0.40	0.24–0.64	0.39	0.24–0.62
≧ 13	1.33	0.75–2.35	1.25	0.71–2.19
**Marriage**				
Yes	0.80	0.61–1.04	0.78	0.60–1.00
No	1		1	
**Smoking**				
Yes	0.67	0.43–1.03	0.67	0.44–1.02
No	1		1	
**Drinking**				
Yes	0.92	0.63–1.33	0.89	0.62–1.28
No	1		1	
**Hypertension**				
Yes	1.08	0.84–1.40	1.11	0.87–1.43
No	1		1	
**Diabetes**				
Yes	1.72	1.25–2.36	1.74	1.27–2.40
No	1		1	
**Heart disease**				
Yes	0.93	0.70–1.23	0.92	0.70–1.22
No	1		1	
**Stroke**				
Yes	2.00	1.32–3.03	2.19	1.45–3.32
No	1		1	
**Cancer**				
Yes	1.16	0.64–2.10	1.14	0.64–2.04
No	1		1	
**Tea consumption**				
< 3	0.90	0.67–1.22	0.90	0.68–1.22
≧ 3	1		1	
**Depressive symptoms**				
Yes	1.56	1.21–2.03	1.67	1.28–2.17
No	1		1	
**Life satisfaction**				
Satisfied	0.90	0.70–1.16	0.87	0.68–1.12
Unsatisfied	1		1	

## Discussion

This study examined the combined effect of PA and F&V intake on cognitive decline in older Taiwanese individuals. When PA and F&V intake were combined, the simultaneity of high PA and F&V intake significantly reduced the risk of cognitive decline by up to 63%. Independently, high PA and F&V intake were associated with a decreased risk of 60% and 40% cognitive decline. Presently, PA and F&V intake are widely recommended health behaviors. F&V intake has been independently proven to reduce the risk of diabetes, stroke, heart disease, and cognitive functional decline^47–50^. The relationship between PA and cognitive function has mainly been explored in older adults in the US^51,52^, and knowledge on the combined effect of PA and F&V intake on cognitive function in older Asian adults is very limited.

Our study adds significantly new knowledge on aging and cognitive decline in two areas. First, the results support the essential findings of a combined effect of PA and F&V intake on cognitive decline in Taiwan. The findings also show that PA and F&V intake play a vital role in improving the cognitive functions of older Taiwanese. PA and F&V intake were important predictors of cognitive decline. The longitudinal data empirically demonstrated the hypothesized inverse relationship of these two health promotion actions (PA and F&V intake) to cognitive function in middle-aged and older Taiwanese adults. Therefore, our analysis of the longitudinal data obtained from Taiwan has further confirmed the importance of simultaneity in PA practice and F&V intake that can attenuate the risk of cognitive decline. Practicing these habits/behaviors simultaneously yields more significant health benefits than practicing them alone. Herein, F&V intake was not independently associated with cognitive decline. Compared with that when high PA and F&V intake were assessed separately, the risk of cognitive decline decreased by 63% when high PA and F&V intake were combined. Unlike Western countries with more PA habits and wellness facilities, Asians have established fewer PA habits owing to insufficient facilities^53^.

This pioneering study explored the 16-year longitudinal relationship between PA and F&V intake in middle-aged and older adults in Taiwan. Second, this study reported significant longitudinal results based on a national population-based cohort study of cognitive function in older Taiwanese individuals. Third, GEE models were used to analyze the five waves of panel data during the 16-year follow-up period. The GEE used for the multi-wave data has properly handled auto-correlated data. Furthermore, the main advantage of GEE is its convenient estimation of population-averaged regression coefficients despite possible misspecification of the parameters^45,46^.

Although the mechanism of the positive effect of PA and F&V intake on cognitive decline is unclear, there are several possible mechanisms underlying cognitive decline. PA is a risk factor for cognitive decline; high PA levels are associated with better cognitive performance^54–57^. Our study shows that high PA levels can effectively reduce the risk of subsequent cognitive decline. This finding is consistent with that of many previous studies^58,59^. Daily PA has been found to prevent cognitive decline in older adults and help delay their already present cognitive decline. Different kinds of sports lead to mental stimulation properties, such as the need for eye-hand coordination and visual and spatial memory, which further enhance their impact on cognitive function^60^. In 2018, 26 researchers representing nine countries and various academic disciplines met in Snekkersten, Denmark to reach an evidence-based consensus on PA in older adults. They reached a consensus on the effects of PA on fitness, health, cognitive function, functional capacity, engagement, motivation, psychological well-being, and social inclusion. Studies have also suggested that the effect of exercising on dementia may be related to the stimulation of neurotrophic factors (brain-derived neurotrophic factors) secreted by the brain, which can prevent the hippocampus from shrinking and maintain cognitive function^10–12,61^. PA can help promote the activities of five senses such as sight, hearing, taste, touch and smell^62^. Simultaneously, proper PA is also helpful in preventing dementia and restoring cognitive function, which is consistent with the literature.

Our panel data analysis revealed that a high F&V intake was an important factor in significantly reducing the risk of cognitive decline. Recently published integrated analyses have consistently found that increased F&V intake is associated with decreased risk of cognitive decline. Our findings are in line with the findings indicating that diet plays an important role in cognitive decline. Foods rich in antioxidants, such as fruits, vegetables, and nuts can prevent or delay cognitive decline. A higher F&V intake reduces or prevents cognitive decline^50,63^. Many known protective mechanisms exist, such as polyphenolic compounds in many plant foods, and bioactive compounds in various fruits, vegetables, legumes, nuts, and whole grains, including antioxidants, vitamins, and polyphenols. Reducing oxidation, other phytochemicals, and unsaturated fatty acids can enhance synaptic plasticity and neuron survival^64,65^, alleviate cognitive decline, and improve cognitive health and specific cognitive areas (especially frontal lobe executive function). Polyphenols in fruits and vegetables can regulate tau hyperphosphorylation and beta-amyloid aggregation in animal models of Alzheimer’s disease^66,67^. Taken together, the different nutrients contained in fruits and vegetables can reduce the risk of cognitive decline in middle-aged and older adults. Third, this study also had a multiplier effect on cognitive decline by combining high F&V intake with high PA, reducing the risk of dementia by 67%. Furthermore, additional evidence is needed to demonstrate a dose–response relationship that could strengthen and support the rationale for practicing the combined PA and F&V intake to mitigate the risk of cognitive decline.

The explanation of the effects of PA and F&V intake on cognitive function or decline is attributable to neuroscience discoveries. For instance, personal and situational factors may change during aging. Elderly individuals also understand that they experience dynamic changes in physical, mental, and social functions. If dietary change is coupled with the development of PA habits simultaneously, the effectiveness of consuming an adequate amount of fruits and vegetables could help achieve an optimal health status and prevent cognitive decline^68–70^. Because young urbanites are busy at work, older adults in Taiwan and other Asian countries generally have poor habits, such as imbalanced eating and lack of exercise, and the risk of chronic diseases in their later years increases^71^. Because the human body deteriorates with age, both PA and F&V intake are healthy behaviors that require continuity and discipline; therefore, a healthy attitude towards PA and F&V intake is essential. According to Harooni et al.^72^, regular healthy living habits and discipline are vital for middle-aged and older adults. Practicing both PA and F&V intake requires regular and disciplined maintenance to achieve sound health effects, generate a healthy and successful aging process, and prevent cognitive decline.

PA and nutrition have complementary effects if they are concomitantly included in daily routine. However, if they are not practiced jointly, the effectiveness of the proactive health behaviors for enhancing cognitive functions will be lessened. Based on the results of a survey in Taiwan, the most common PA is walking (69.6%), gymnastics (14.9%), and hand-shaking moments (8.5%). Noteworthy, Hand-shaking as part of the body and mind exercise with varying forms can facilitate the blood circulation and muscle relaxation while one stretches and moves the arms up and down. It is a popular movement for Qigoing (Chi-Kung). For example, Dr. Li, a founder of the Chinese Biological Energy Medical Qigong, has authored the Seventeen-Steps-of Physical Wellness and documented health benefits of hand-shaking moments as part of the Chi-Kung exercise that will enhance the blood circulation and stimulate the growth of energy. Evidence based on the medical Qigong and Tai-chi also shows that cognitive function improvement is related to the Chi-Kung practice, including hand-shaking moments^73,74^. PA with low intensity and resistance yielded less health-promoting effects; most elderly individuals were engaged in a more specific PA in parks or schools near their homes, and the intensity of PA was insufficient^75,76^. Nutrient supplements with fruits and vegetables are needed to reduce the risk of cognitive decline. Conversely, although a significant amount of F&V intake can increase the nutritional content without PA, the risk of cognitive decline will reduce by 40% and 23%, respectively^77^.

This longitudinal study had some limitations. Cognitive decline was not diagnosed by physicians but was self-reported by the respondents. Although self-reporting has an acceptable validity and accuracy, it inevitably has some limitations. The cognitive status was based on the Chinese version of the SPMSQ, which has been widely used in domestic studies, and its reliability and validity have been confirmed. This study used a relatively long period of observation (16 years), but the assessment of health outcomes and their predictors was not based on the annual survey of the panel (i.e., the consecutive years). Should the annually assessed data be available, we could perform a more rigorous analysis such as latent growth curve modeling of the determinants of cognitive decline^78^. Thus, the trajectories of cognitive function change of the elderly could be better portrayed.

Dietary activity and PA may change with age, health status, social environment, and other factors. Further, the participants were elderly Taiwanese individuals; thus, the results may not be generalizable to younger Taiwanese individuals. Data relevant to biomedical markers should also be collected in panel studies on aging, and the quantity and quality of F&V intake should be further assessed. A randomized clinical trial could be designed and implemented for varying older adult groups so that the precise dose–response relationship between the intensity of a proposed intervention, the combined effect of PA and F&V intake, and cognitive decline could be further demonstrated.

## Conclusions

The relationship between PA and F&V intake and cognitive decline in older adults was investigated via a longitudinal study in Taiwan. This study confirmed the beneficial effects of the combined PA practice and F&V intake on cognitive decline. High F&V intake and PA level can effectively reduce the risk of cognitive decline and help maintain good long-term health among elderly individuals in Taiwan. For middle-aged and older adults, changes owing to aging and the environment may restrict their physical function (e.g., tolerance, cardiopulmonary function, muscle strength, and mobility). Therefore, it is imperative to develop a standardized and specific protocol for promoting healthy habits, such as PA and F&V intake, in different elderly groups. We also strongly advocate that older adults should have an adequate amount of regular PA practice every day and prepare a specific amount of F&V intake for each meal. These personal actions are safe, effective, and economical approaches to health promotion and disease prevention.

## Data Availability

The data that support the findings of this study are available from the Health Promotion Administration of the Department of Health & Welfare of Taiwan but restrictions apply to the availability of these data, which were used under license for the current study, and so are not publicly available. Data are however available from the authors upon reasonable request and with permission of the Health Promotion Administration of the Department of Health & Welfare of Taiwan. The study clarified that the data used in this study was anonymized. Ethical approval was obtained from Institutional Review Board of Tri-Service General Hospital, National Defense Medical Center (TSGHIRB No. B-109-30).
